# Validation study of a nomogram for predicting probability of low risk of MammaPrint results in women with clinically high-risk breast cancer

**DOI:** 10.1007/s12672-022-00604-z

**Published:** 2022-12-23

**Authors:** Young Sol Hwang, Hwa Jung Kim, Jisun Kim, Il Yong Chung, Beom Seok Ko, Hee Jeong Kim, Jong Won Lee, Byung Ho Son, Sei-Hyun Ahn, Sae Byul Lee

**Affiliations:** 1 University of Ulsan College of Medicine, Seoul, Republic of Korea; 2 Department of Biostatistics, University of Ulsan, College of Medicine, Asan Medical Center, Seoul, South Korea; 3 Department of Surgery, University of Ulsan, College of Medicine, Asan Medical Center, 88 Olympic-ro 43-gil, Songpa-gu, Seoul, 138-736 South Korea

**Keywords:** Breast cancer, 70 gene signature, Nomogram, Validation, Disease-free survival

## Abstract

**Background:**

MammaPrint (MMP) helps clinicians identify the ideal time for adjuvant treatment for patients with early HR+/HER2− breast cancer. We aimed to validate a nomogram designed to predict probability of low risk of MMP results and to evaluate the difference in survival outcome between two groups divided by nomogram score.

**Methods:**

In this retrospective cohort study, we evaluated 172 patients from Asan Medical Center, Seoul, Korea, who underwent breast cancer surgery and MMP during 2020–2021. First, we validated the nomogram by calculating the area under the curve (AUC) and using calibration. Additionally, with the data of 1,835 T1-3N0-1M0 HR+/HER2− patients from Asan Medical Center during 2010–2013, we compared the disease-free survival (DFS), overall survival (OS), and breast cancer-specific survival (BCSS) rates by Kaplan−Meier analysis between the two groups divided by nomogram total point (TP) of 183.

**Results:**

The AUC calculated by validation of 172 patients was 0.73 (95% confidence interval [CI], 0.66–0.81). The discrimination and calibration of the prediction model were satisfactory following additional validation of 1835 patients. The high-risk and low-risk groups had different 5-year OS (97.9% vs. 98.1%, *p* = 0.056), DFS (98.6% vs. 99.4%, *p* = 0.008), and BCSS rates (98.6% vs. 99.4%, *p* = 0.002).

**Conclusion:**

For treatment decision-making among clinically high-risk patients with HR+/HER2− and node-positive disease, the nomogram showed satisfactory performance in predicting patients with low genomic risk. Survival outcome significantly differed between two groups divided by nomogram TP. More studies are needed to validate this model in international cohorts and large prospective cohorts from other institutions.

**Supplementary Information:**

The online version contains supplementary material available at 10.1007/s12672-022-00604-z.

## Introduction

The current treatment approach for hormone receptor-positive and human epidermal growth factor receptor 2 (HER2)-negative breast cancer patients is focused on selecting patients who can be spared chemotherapy [1–4]. Previously there were no tools for classifying patients who needed chemotherapy and those who did not. However, there have been several attempts to assess breast cancer patients’ risk with genomic analysis; finally, several guidelines advise using genomic assay results to identify people needing chemotherapy [5–8]. The National Comprehensive Cancer Network (NCCN) guidelines for breast cancer recommend using MammaPrint(MMP) for decision-making for chemotherapy in patients with N0-1 early-stage breast cancer [9]. While Oncotype DX is widely used in patients who have no metastasis to lymph nodes, MMP test has higher level of evidence for node-positive luminal A breast cancer patients [9].

Use of genomic analysis for risk assessment is key in treatment decision-making for breast cancer; however, there are some disadvantages to using this new tool. In the medical setting in Korea, national health insurance does not cover the cost of genomic analysis, thus imposing burdens on patients who already pay for the breast surgery. Furthermore, the time taken to obtain results of genomic tests is long, and patients are unable to receive therapy until the test results are obtained. For these reasons, prediction of MMP recurrence score in advance, using clinicopathological data, may be useful. In our previous study, we created a nomogram that can easily predict the MMP risk score as high or low using 4 clinicopathologic features: patient age, progesterone receptor status (PR), nuclear grade, and Ki-67 [10]. Data on these four factors are easily obtained in clinical settings. Although this nomogram is useful in predicting patient prognosis, it is still hard to correlate the result from the nomogram with survival benefit. Additionally, the size of the validation group in the previous study was insufficient considering that treatment choice is critical for patient outcome.

As the nomogram needs further validation, we have used recent patient data from Asan Medical Center, Seoul, to do so. We also used HR+/HER2− breast cancer patient data from 2010 to 2013, which has patient recurrence and survival data with clinicopathologic data, to investigate whether the nomogram can identify people with survival benefit.

## Methods

### Patients

Validation Set 1 enrolled breast cancer patients who were T1-3N0-1M0 hormone receptor-positive and HER2-negative who underwent MMP who had breast cancer surgery between 2020 and 2021 at Asan Medical Center, Seoul, Korea. The dataset of 172 cases who were eligible was used for validation of the nomogram created in our previous study that predicts MMP results using clinical data including age, nuclear grade, PR status, and Ki-67 results [10]. Clinical data obtained included patients’ age at surgery, sex, surgery type, TNM stage, adjuvant TNM stage, cancer size, Lymph node (LN) status, histologic and nuclear grade, Lymphovascular invasion (LVI), Hormone receptor status, HER2 status, Ki-67, and p53. Nuclear staining for ER and PR was evaluated using the Allred scoring method (0–8). Membrane staining for HER2 was evaluated using the HercepTest (BenchMark XT autostainer using OptiView DAB Detection Kit, Ventana Medical Systems, Tucson, AZ, USA) protocol. Immunohistochemistry for Ki-67 (1:250, MIB-1, Dako, Glostrup, Denmark) was performed using a BenchMark XT autostainer (Ventana Medical Systems) with an i-View detection kit (Ventana Medical Systems). Pathologic staging was determined based on the American Joint Committee on Cancer Staging Manual 7th edition.

Further, we used Validation Set 2, 1835 T1-3N0-1M0 HR+/HER2− patients from Asan Medical Center from 2010 to 2013, which included survival data and recurrence data, to analyze whether the nomogram can be associated with survival benefit identification as well. We compared the survival outcomes of two groups divided by nomogram score. The outcomes were disease-free survival (DFS), overall survival (OS), and breast cancer-specific survival (BCSS). DFS was defined as the time from the date surgery to the first date of disease recurrence; OS, the time from the date of surgery to the date of a patient’s death from any cause; BCSS, the time from the date of surgery to the date of a patient’s death from breast cancer. This study was reviewed and approved by the Institutional Review Board of Asan Medical Center (2017-1341). Informed consent was waived because the study was based on retrospective clinical data.

### Statistical analysis

For validation, the MMP results of 172 cases were used. The four factors that were significant according to the nomogram, age at diagnosis (20–100), nuclear grade (range, 1–3), Allred scores of PR status (range, 0–8) and Ki-67 labeling index (0-100), were used to validate nomogram predictability. The Chi-square and Fisher’s exact tests were used for between-group comparisons of clinicopathological characteristics, based on MMP results. We conducted a robustness analysis to validate our model and employed receiver operating characteristic (ROC) analysis and calculated the area under the curve (AUC). Using the Kaplan-Meier method to further validate our nomogram system, we generated survival curves for breast cancer patients from 2010 to 2013. The significance of differences in survival was tested using the log-rank test. All data analyses were performed using R statistical package ver 3.2.0 (http://r-project.org). Significance level was set at 0.05, and all p values were two sided.

## Results

### Patient characteristics

Detailed information on patient characteristics of the Validation Set 1 cohort (n = 172) classified into MMP score high-risk group versus MMP score low-risk group is found in Table 1. The number of patients with initial T stage 1, 2, and 3 were 79 (45.9%), 89 (51.7%), and 4 (2.4%), respectively. Approximately 97.7% patients had lymph node involvement (n = 168). The mean age at initial operation for the entire cohort was 52.0 ± 10.3 years. There were no patients with ER Allred negative or weak positive scores; 4 (2.3%) had intermediate and 168 (97.7%) had strong positive scores. As regards PR Allred score, there were 16 (9.3%) patients with negative; 17 (9.9%), weak; 37 (21.5%), intermediate; and 102 (59.3%), strong positive scores. Approximately 43.0% of patients had positive LVI, and 47.1% showed high Ki-67. Additional file 1: Table S1 shows the dataset of the 407 patients used for developing the nomogram in the previous study.

**Table 1:** Comparison between the characteristics of MMP low-risk and MMP high-risk patients with those of the total validation set 1 patient cohort

Variables	Total	MMP low	MMP high	*p* value
N	172	93	79	
Age at diagnosis (yr) (mean ± SD)	52.0 ± 10.3	52.2 ± 9.9	51.8 ± 10.8	0.829
Histological grade				< 0.001
Grade I	8 (4.7)	7 (7.5)	1 (1.2)	
Grade II	137 (79.6)	83 (89.2)	54 (68.4)	
Grade III	27 (15.7)	3 (3.3)	24 (30.4)	
Nuclear grade				< 0.001
Grade I	2 (1.2)	1 (1.1)	1 (1.2)	
Grade II	143 (83.1)	89 (95.7)	54 (68.4)	
Grade III	27 (15.7)	3 (3.2)	24 (30.4)	
Estrogen receptor				0.238
Negative	0 (0.0)	0 (0.0)	0 (0.0)	
Weak	0 (0.0)	0 (0.0)	0 (0.0)	
Intermediate	4 (2.3)	1 (1.1)	3 (3.8)	
Strong	168 (97.7)	92 (98.9)	76 (96.2)	
Progesterone receptor				0.097
Negative	16 (9.3)	6 (6.5)	10 (12.7)	
Weak	17 (9.9)	7 (7.5)	10 (12.7)	
Intermediate	37 (21.5)	17 (18.3)	20 (25.3)	
Strong	102 (59.3)	63 (67.7)	39 (49.3)	
Lymphovascular invasion				0.215
Negative	98 (57.0)	57 (61.3)	41 (51.9)	
Positive	74 (43.0)	36 (38.7)	38 (48.1)	
p53				0.10
0	17 (9.9)	7 (7.5)	10 (12.7)	
1	78 (45.3)	52 (55.9)	26 (32.9)	
2	65 (37.8)	31 (33.3)	34 (43.0)	
3	12 (7.0)	3 (3.3)	9 (11.4)	
Ki-67 level				< 0.001
Low Ki-67 < 20%	92 (52.9)	63 (67.7)	28 (35.4)	
High Ki-67 ≥ 20%	81 (47.1)	30 (32.3)	51 (64.6)	
Breast surgery				0.932
Total mastectomy	55 (32.0)	30 (32.3)	25 (31.6)	
Breast conservation surgery	117 (68.0)	63 (67.7)	54 (68.4)	
Axillary operation				0.984
Sentinel node biopsy	63 (36.6)	34 (36.6)	29 (36.7)	
Axillary dissection after sentinel node biopsy	109 (63.4)	59 (63.4)	50 (63.3)	
T stage				0.693
T1	79 (45.9)	42 (45.2)	37 (46.8)	
T2	89 (51.7)	48 (51.6)	41 (51.9)	
T3	4 (2.4)	3 (3.2)	1 (1.3)	
N stage				0.238
N0	4 (2.3)	1 (1.1)	3 (3.8)	
N1	168 (97.7)	92 (98.9)	76 (96.2)	
Stage				0.693
Stage I	2 (1.2)	1 (1.1)	1 (1.2)	
Stage II	166 (98.5)	89 (95.7)	77 (97.6)	
Stage III	4 (2.3)	3 (3.2)	1 (1.2)	
Tumor size (cm) (mean ± SD)	2.37 ± 1.13	2.4 ± 1.3	2.4 ± 0.9	0.878
Number of positive nodes				0.273
0	3 (1.7)	0 (0.0)	3 (3.8)	
1	110 (64.0)	59 (63.4)	51 (64.6)	
2	48 (27.9)	28 (30.1)	20 (25.3)	
3	11 (6.4)	6 (6.5)	5 (6.3)	
Largest positive node size (mm)	6.23 ± 4.81	6.0 ± 4.4	6.5 ± 5.3	0.441

### Model predicting MMP results and validation of nomogram with validation set 1

Of the 172 patients, 93 (54.1%) were MMP low and 79 (45.9%) were MMP high. The mean age at diagnosis among those with MMP low was 52.1 $\pm$ 9.9 years; for MMP high, it was 51.8 $\pm$ 10.8 years (*p *= 0.829). The MMP high group had higher histologic and nuclear grades, all with *p *< 0.001, compared to the MMP low-risk group. There were 64.6% patients with high Ki-67 level in the MMP high group, compared to 32.3% in the MMP low group.

As regards estrogen receptor status, most patients showed strong Allred scores (7–8). Only 1 (1.1%) patient in the MMP low-risk group and 3 (3.8%) in the MMP high-risk group had intermediate Allred score (5–6). No patients showed negative or weak positive ER Allred score. PR status showed distinct characteristics compared to that of ER status; however, these were not significant. No difference was found in the presence of LVI, p53 status, and surgical methods. Pathologic stages of MMP low and high, tumor size, and number of positive nodes and largest positive node size showed no statistical relevance.

**Fig. 1:**
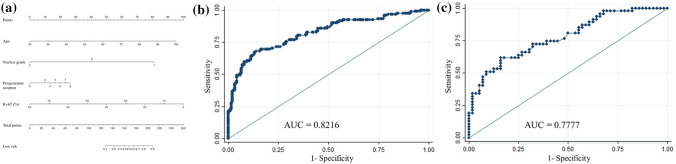
**a** Nomogram to predict low-risk recurrence score of MammaPrint result and receiver operating characteristic curve of nomogram. **b** Training group of 306 patients. **c** Validation group of 103 patients

**Fig. 2:**
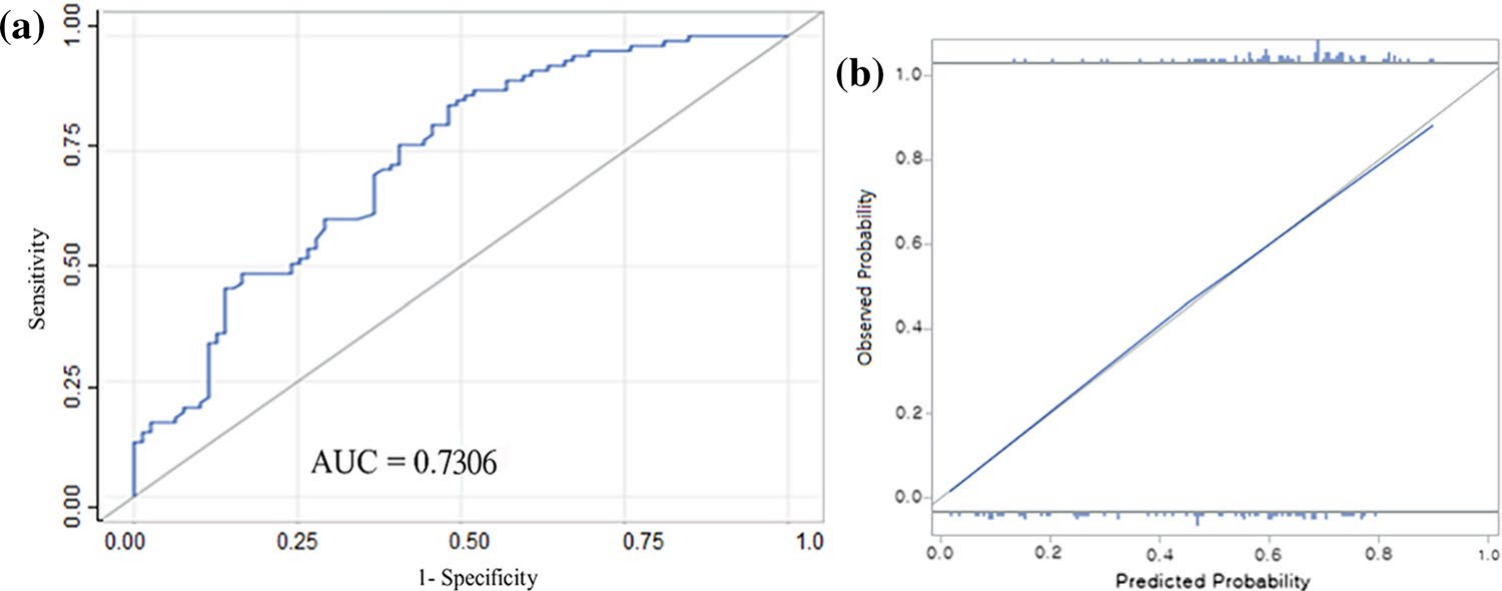
**a** Receiver operating characteristic curve of nomogram. Validation cohort of 172 patients. **b** Calibration plot of nomogram

The nomogram (Fig. 1a) was initially made by training set and later validated by validation set, and the AUC was 0.82 (95% CI, 0.77–0.87) (Fig. 1b) and 0.77 (95% CI, 0.68–0.86) (Fig. 1c), respectively [10]. To validate the nomogram, we used the patient cohort of 172 breast cancer patients who underwent MMP testing. The AUC of the Validation Set 1 was 0.73 (95% CI, 0.66–0.81) (Fig. 2). The calibration plot (Fig. 2) shows good calibration.

### Additional validation of the nomogram with an independent cohort

We performed an additional validation study with the data of 1,835 T1-3N0-1M0 HR+/HER2- patients, Validation Set 2. The patient cohort was classified into two groups, low-risk and high-risk group, based on a nomogram value of 183. A cutoff of 183 was selected based on Youden index, which consider both sensitivity and specificity to find optimal cutoff value. Detailed characteristics of the patient cohort used for additional validation (n = 1,835) can be seen in Table 2. The patient’s cohort data is divided into two groups by Total point (TP) cutoff of 183. The mean age at diagnosis was 44.7$\pm$9.0 years vs. 50.6 $\pm$ 9.2 years in TP<183 and TP $\ge$ 183 groups, respectively (p < 0.001). The TP < 183 group had a higher histologic grade, a higher nuclear grade, and a higher Ki-67 level and p53 than the low-risk group (p < 0.001). ER (p = 0.029) and PR (p < 0.001) status were different between the two groups. The TP<183 group had 89 (13.2%) and 572 (85.2%) with intermediate and strong ER Allred scores, respectively. On the other hand, the TP $\ge$ 183 group had 115 (9.9%) and 1039 (89.3%) with intermediate and strong ER Allred scores, respectively, which indicated higher percentage of strong ER Allred score. That of PR was also similar, where 194 (28.9%) vs. 210 (18.1%) patients had intermediate PR Allred score, and 412 (61.3%) vs. 913 (78.5%) had strong PR Allred score. Pathologically confirmed results showed higher stages in T and N stages in the TP<183 group. In the TP<183 group, only 401 (59.7%) had T1 disease and 459(68.3%) had N0; on the contrary, there were 857 (73.7%) patients with T1 stage disease and 848 (72.9%) with N1 stage in the TP $\ge$ 183 group.

**Table 2:** Characteristics of the validation set 2 patient cohort

Variables	Total	TP < 183	TP $\ge$ 183	*p* value
N	1835	672	1163	
Age at diagnosis (Mean ± SD)	48.5 ± 9.6	44.7 ± 9.0	50.6 ± 9.2	< 0.001
Histological grade				< 0.001
Grade I	106 (5.8)	3 (0.4)	103 (8.9)	
Grade II	1472 (80.2)	412 (61.3)	1060 (91.1)	
Grade III	257 (14.0)	257 (38.3)	0 (0.0)	
Nuclear grade				< 0.001
Grade I	94 (5.1)	2 (0.3)	92 (7.9)	
Grade II	1469 (80.1)	412 (61.3)	1057 (90.9)	
Grade III	272 (14.8)	258 (38.4)	14 (1.2)	
Estrogen receptor				0.029
Negative	1 (0.1)	1 (0.1)	0 (0.0)	
Weak	19 (1.0)	10 (1.5)	9 (0.8)	
Intermediate	204 (11.1)	89 (13.2)	115 (9.9)	
Strong	1611 (87.8)	572 (85.2)	1039 (89.3)	
Progesterone receptor				< 0.001
Negative	0 (0)	0 (0)	0 (0)	
Weak	106 (5.8)	66 (9.8)	40 (3.4)	
Intermediate	404 (22.0)	194 (28.9)	210 (18.1)	
Strong	1325 (72.2)	412 (61.3)	913 (78.5)	
Lymphovascular invasion				< 0.001
Negative	1447 (78.9)	484 (72.0)	963 (82.8)	
Positive	388 (21.1)	188 (28.0)	200 (17.2)	
p53				< 0.001
0	1144 (62.3)	365 (54.3)	779 (67.0)	
1	367 (20.0)	110 (16.4)	257 (22.1)	
2	161 (8.8)	78 (11.6)	83 (7.1)	
3	163 (8.9)	119 (17.7)	44 (3.8)	
Ki-67 level				< 0.001
Low Ki-67 < 20%	891 (48.5)	88 (13.1)	803 (69.0)	
High Ki-67 ≥ 20%	944 (51.5)	584 (86.9)	360 (31.0)	
Breast surgery				0.001
Total mastectomy	511 (27.8)	218 (32.4)	293 (25.2)	
Breast conservation surgery	1324 (72.2)	454 (67.6)	870 (74.8)	
Axillary operation				0.006
Axillary dissection	38 (2.1)	21 (3.1)	17 (1.5)	
Sentinel node biopsy	1439 (78.4)	504 (75.7)	935 (81.0)	
Axillary dissection after sentinel node biopsy	343 (18.7)	141 (21.2)	202 (17.5)	
T stage				< 0.001
T1	1258 (68.6)	401 (59.7)	857 (73.7)	
T2	542 (29.5)	256 (38.1)	286 (24.6)	
T3	35 (1.9)	15 (2.2)	20 (1.7)	
N stage				0.036
N0	1307 (71.2)	459 (68.3)	848 (72.9)	
N1	528 (28.8)	213 (31.7)	315 (27.1)	
Stage				< 0.001
Stage I	1048 (57.1)	318 (47.3)	730 (62.8)	
Stage II	763 (41.6)	346 (51.5)	417 (35.8)	
Stage III	24 (1.3)	8 (1.2)	16 (1.4)	
Chemotherapy				< 0.001
No	1084 (59.0)	335 (49.9)	749 (64.5)	
Yes	748 (40.8)	335 (49.9)	413 (35.4)	
Unknown	3 (0.2)	2 (0.2)	1 (0.1)	
Radiotherapy				0.031
No	477 (26.0)	194 (29.0)	283 (24.4)	
Yes	1355 (73.8)	476 (70.9)	879 (75.5)	
Unknown	3 (0.2)	2 (0.1)	1 (0.1)	
Anti-hormonal therapy				0.547
No	21 (1.1)	9 (1.2)	12 (0.9)	
Yes	1809 (98.6)	660 (98.7)	1149 (99.0)	
Unknown	5 (0.3)	3 (0.1)	2 (0.1)	

*SD* standard deviation

**Fig. 3:**
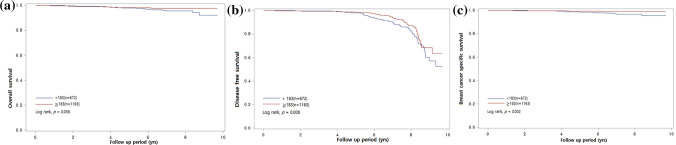
Kaplan-Meier analysis of validation set 2 according to the cutoff of 183. **a** Overall survival. **b** Disease-free survival, and **c** Breast cancer-specific survival

To assess the efficacy of the nomogram in defining the prognosis of the patients, Kaplan-Meier analysis was used. We identified that the high-risk group according to the nomogram had significantly lower DFS (p = 0.008) and 5-year BCSS rates (98.6% vs. 99.4%, *p* = 0.002) compared to the low-risk group (Fig. 3), In the case of 5-year OS (98.1% vs. 97.9%, *p* = 0.056), the high-risk group showed inferior survival trend compared to the low-risk group. Thus, the nomogram can indeed distinguish the better-prognosis group from the worse-prognosis group.

## Discussion

For patients with early-stage breast cancer, predicting the response to chemotherapy and risk of recurrence is crucial. Genomic testing for early breast cancer is a reliable tool for decision making for treatment. For adjuvant systemic therapy in patients with non-metastatic, ER/PR positive, HER2 negative, and N0-1 breast cancers, the 70-gene MMP test is recommended as evidence of category 1 in the 2022 NCCN guidelines [9]. Moreover, the St. Gallen Consensus Conference, American Society of Clinical Oncology (ASCO), and European Commission Initiative on Breast Cancer recommended use of the 70-gene signature in women with high clinical risk factors [7, 11, 12]. Therefore, the 70-gene signature test is increasingly known for its usefulness in determining candidates for adjuvant chemotherapy. Although there is evidence for the usefulness of MMP in certain subsets of patients, clinicians hesitate to recommend this test because of the cost and long duration to obtain test results.

The nomogram in our previous study helped clinicians promptly predict a low MMP risk, using only four simple clinicopathologic factors (age, nuclear grade, progesterone receptor status, and Ki-67) [10]. According to multivariate analysis, age and progesterone receptor status showed positive relationship with MMP low risk and their regression coefficient were 0.65 (95% confidence interval [CI], 0.32–0.98) and 0.18 (95% confidence interval [CI], 0.05–0.31), respectively. Nuclear grade and Ki-67 had negative association with MMP low risk and their regression coefficient were − 2.22 (95% confidence interval [CI], − 3.75 to − 0.69) and − 0.07 (95% confidence interval [CI], − 1.69 to 0.04). Initially the nomogram was made by cohort of 409 T1-3N0-1M0 hormone receptor-positive and HER2-negative breast cancer patients [10]. The total 409 cohort was randomly classified into two separate set, 306 training set and 103 validation set (Training set was used to build nomogram and validation set was used to validate the nomogram). Other studies have attempted to predict the MMP score by using the MR imaging radiomics signature or other clinicopathologic data [13, 14]. However, we decided that the easiest way to make a quick estimation is by nomogram which is ran by simple four simple factors [10]. On top of it we added user-friendly interfaced calculator that can calculate the MMP score in a second.

In this retrospective cohort study, which aimed to validate a nomogram that can predict the probability of a low risk of MammaPrint results in women with clinically high-risk breast cancer (according to definition in Adjuvant! Online), we found out the AUC of Validation Set 1 was 0.73 (95% CI, 0.66–0.81). In order to further validate the nomogram by seeking correlation of low risk of MammaPrint and long-term survival of patients, additional study was done with Validation Set 2. Kaplan–Meier analysis of the two groups divided by a nomogram score of 183 showed a relevant difference in OS (*p* = 0.056), DFS (*p* = 0.008) and BCSS (*p* = 0.002). Though we have chosen 183 as an ideal cutoff for discriminating low-genomic risk and the basis for the choice of treatment option(Sensitivity and specificity is 84% and 65%, respectively), however, clinicians can choose another cutoff, if their primary preference is another combination of sensitivity and specificity. In MINDACT trial, the primary outcome was to assess the presence of non-inferiority in 5-year survival without distant metastasis, among patients who have high-risk clinical features and did not receive chemotherapy, but have low genomic risk score [15]. Similarly, our study investigated whether nomogram score, which was originally designed to find out low risk of MMP, can be related to survival advantage in high clinical risk patients. As low risk in MMP is conventionally thought of as sign of good prognosis, we have tried to verify the association of nomogram score with survival prediction ability.

On closely inspecting the cohort profiles of 1,835 patients at AMC from 2010 to 2013, we found that no patient in the group with TP $\ge$ 183 had histologic grade III, while the group with TP<183 had 257(38.3%) patients with grade III. In addition, the groups with TP $\ge$ 183 and TP < 183, had 103 (8.9%) and 3 (0.4%) breast cancer patients with grade I, respectively. Since it was first demonstrated in 1991 [16], the histologic grade is a well-known prognostic factor in breast cancer. Consensus and recommendations for good practice in determining the tumor grade set by pathology in breast cancer [17] render the tumor grade a simple and accurate determinant in decision making regarding adjuvant therapy. Despite developments in new molecular technology, tumor grade determination remains a validated method for assessing patient prognosis when alternative molecular testing is not available [18].

Age at diagnosis plays a key role in our model; younger patients (< 50 years) are unlikely to have 95% or higher low genomic risk probability according to the nomogram calculator [10]. In our study, there was a considerable difference in the mean age at diagnosis in the Validation Set 2 (44.7 ± 9.0 years in the high-risk group and 50.6 ± 9.2 years in the low-risk group). Most recently updated results of the MINDACT trial, with a median follow-up of 8.7 years, reveal that in clinically high/genomic low risk patients, women aged 50 years or younger have a relevant difference in 8-year distant metastasis-free survival, based on the type of adjuvant therapy administered [19]. The absolute difference in the 8-year distant metastasis-free survival in the chemotherapy and no-chemotherapy groups were 5.0 and 0.2% point respectively, and this result might be the consequence of ovarian function suppression effect of chemotherapy which only affect premenopausal women. Our study result corresponds with up-to-date interest in age at diagnosis, which is considered crucial prognostic factor in the selection of adjuvant treatment options.

In terms of disease-free survival, the TP $\ge$ 183 group showed significantly superior outcome over the TP<183 group (p = 0.008). HR+ and HER2− breast cancers are reported to have 20% likelihood of tumor recurrence in the first 10 years after surgery, although many patients do not experience recurrence [20]. During the first few years of adjuvant endocrine therapy [21], patients with a high clinical risk and/or pathologic features have a higher risk of disease recurrence. According to the monarchE phase III trial, distant metastasis accounts for more than 75% of early recurrences during endocrine therapy [22]. Therefore, optimizing adjuvant therapy according to patient specific risk is important for improving invasive disease-free survival. After reviewing the limitations of our previous study, the nomogram was constructed based on the MMP result, but we did not consider the treatment outcome of a patient who received adjuvant therapy. This study aimed to provide evidence of the possibility that the nomogram could help distinguish the groups with favorable and worse outcomes. Moreover, by validating the nomogram using a patient cohort from another time period, 2010 to 2013, this study strengthens the usability of the nomogram.

There are some limitations that could be found into this study. First, this study is based on a retrospective analysis of patient datasets and, therefore, there might have been selection bias in selecting the patient cohort. Validation Set 1 and 2 show different patients characteristics, because set 1 only enrolled patients selected for costly MMP test and, therefore, Validation Set 1 had few ER-PR, negative, or weak positive patients, leading to inaccurate predictions in the ER-PR negative or weak positive groups. However, this aspect can also be a strong point of this study, because in real-world clinical settings, most ER-PR negative or weak positive patients are omitted from MMP tests. In addition, as Ki-67 levels may vary between institutions, there can be issues with reproducibility in other institutions. Therefore, further validation studies using cohorts from other institution may help confirm the usability of this nomogram. Mistranslation of the nomogram results into overall prognosis of individual patients by other clinicians is also a concerning issue. Due to its retrospective nature, this study is incapable of showing the predictive value of this test. Moreover, the validation of nomogram was conducted by the cohort of one institute, therefore, the usability of nomogram could be later validated by multi-institutional analysis.

In conclusion, for decision making regarding treatment in HR+/HER2− and node positive, clinically high-risk patients, our nomogram is useful for quick prediction of low genomic risk patients who can be spared MMP testing. In addition, the two groups distinguished by the nomogram score differed in the 5-year DFS and BCSS, and OS. Further studies are needed to validate this model in international cohorts and large prospective cohorts from other institutions.

## Supplementary Information

**Additional file 1:** **Table S1.** Comparison of characteristics of MMP low-risk and high-risk patients in the validation and training sets.

## Data Availability

All data generated or analysed during this study are included in this published article (and its supplementary information files).
