# Supplementary material for: Validation study of a nomogram for predicting probability of low risk of MammaPrint results in women with clinically high-risk breast cancer
Source: Discov Oncol. 2022 Dec 23;13:141. doi: 10.1007/s12672-022-00604-z (PMC9789221; doi:10.1007/s12672-022-00604-z)
Supplement: Supplementary file 1 — Additional file 1: Table S1. Comparison of characteristics of MMP low-risk and high-risk patients in the validation and training sets. [file 12672_2022_604_MOESM1_ESM.docx]

Additional file 1

Table S1. Comparison of characteristics of MMP low-risk and high-risk patients in the validation and training sets

|  | Validation set | | Training set | |
| --- | --- | --- | --- | --- |
| Variables | MMP low risk | MMP high risk | MMP low risk | MMP high risk |
| N | 103 | | 306 | |
| Age at diagnosis (mean ± SD^*^) | 54.3± 10.4 | 49± 8.6 | 53.0± 8.9 | 48.2± 10.3 |
| Histological grade |  |  |  |  |
| Grade I | 6 (10.0) | 1 (2.0) | 13 (7.2) | 2 (1.7) |
| Grade II | 53 (88.3) | 42 (85.7) | 165 (91.7) | 97 (80.8) |
| Grade III | 1 (1.7) | 6 (12.2) | 2 (1.1) | 21 (17.5) |
| Nuclear grade |  |  |  |  |
| Grade I | 1 (1.7) | 0 (0.0) | 1 (0.6) | 0 (0.0) |
| Grade II | 58 (96.7) | 43 (87.8) | 177 (98.3) | 97 (80.8) |
| Grade III | 1 (1.7) | 6 (12.2) | 2 (1.1) | 23 (19.2) |
| Estrogen receptor |  |  |  |  |
| Negative | 0 (0.0) | 0 (0.0) | 0 (0.0) | 0 (0.0) |
| Weak | 0 (0.0) | 0 (0.0) | 0 (0.0) | 0 (0.0) |
| Intermediate | 0 (0.0) | 1 (2.0) | 2 (1.1) | 5 (4.2) |
| Strong | 60 (100.0) | 48 (98.0) | 178 (98.9) | 115 (95.8) |
| Progesterone receptor |  |  |  |  |
| Negative | 6 (10.0) | 9 (18.4) | 10 (5.6) | 12 (10.0) |
| Weak | 2 (3.3) | 5 (10.2) | 10 (5.6) | 20 (16.7) |
| Intermediate | 9 (15.0) | 12 (24.5) | 38 (21.1) | 21 (17.5) |
| Strong | 43 (71.7) | 23 (46.9) | 122 (67.8) | 67 (55.8) |
| Lymphovascular invasion |  |  |  |  |
| Negative | 35 (58.3) | 18 (36.7) | 112 (62.2) | 49 (40.8) |
| Positive | 25 (41.7) | 30 (61.2) | 68 (37.8) | 69 (57.5) |
| p-53 |  |  |  |  |
| 0 | 22 (36.7) | 20 (40.8) | 56 (31.1) | 36 (30.0) |
| 1 | 26 (43.3) | 16 (32.7) | 83 (46.1) | 35 (29.2) |
| 2 | 7 (11.7) | 11 (22.4) | 36 (20.0) | 22 (18.3) |
| 3 | 5 (8.3) | 2 (4.1) | 5 (2.8) | 27 (22.5) |
| Ki-67 level |  |  |  |  |
| low Ki-67 < 20% | 46 (76.7) | 18 (35.7) | 131 (72.8) | 39 (32.5) |
| high Ki-67 ≥ 20% | 14 (23.3) | 31 (63.3) | 49 (27.2) | 81 (67.5) |
| Breast surgery |  |  |  |  |
| Total mastectomy | 21 (35.0) | 11 (22.4) | 48 (26.7) | 33 (27.5) |
| Breast conservation surgery | 39 (65.0) | 38 (77.6) | 132 (73.3) | 85 (70.8) |
| Axillary operation |  |  |  |  |
| Axillary dissection | 1 (1.7) | 0 (0.0) | 2 (1.1) | 0 (0.0) |
| Sentinel node biopsy | 31 (51.7) | 25 (51.0) | 102 (56.7) | 53 (44.2) |
| Axillary dissection after sentinel node biopsy | 28 (46.7) | 24 (49.0) | 75 (41.7) | 65 (54.2) |
| T stage |  |  |  |  |
| T1 | 25 (41.7) | 23 (46.9) | 99 (55.0) | 44 (36.7) |
| T2 | 34 (56.7) | 26 (53.1) | 77 (42.8) | 72 (60.0) |
| T3 | 1 (1.7) | 0 (0.0) | 4 (2.2) | 2 (1.7) |
| N stage |  |  |  |  |
| N0 | 1 (1.7) | 2 (4.1) | 8 (4.4) | 8 (6.7) |
| N1 | 59 (98.3) | 47 (95.9) | 172 (95.6) | 110 (91.7) |
| Stage |  |  |  |  |
| Stage I | 4 (6.7) | 8 (16.3) | 27 (15.0) | 8 (6.7) |
| Stage II | 55 (91.7) | 41 (83.7) | 149 (82.8) | 108 (90.0) |
| Stage III | 1 (1.7) | 0 (0.0) | 4 (2.2) | 2 (1.7) |
| Tumor size (cm) (mean ± SD*) | 2.3± 1.2 | 2.2± 0.9 | 2.1± 1.1 | 2.5± 1.0 |
| Number of positive nodes |  |  |  |  |
| 0 | 1 (1.7) | 0 (0.0) | 8 (4.4) | 8 (6.7) |
| 1 | 32 (53.3) | 36 (73.5) | 118 (65.6) | 69 (57.5) |
| 2 | 23 (38.3) | 9 (18.4) | 43 (23.9) | 36 (30.0) |
| 3 | 4 (6.7) | 4 (8.2) | 11 (6.1) | 5 (4.2) |
| Largest positive node size (mm) | 6.7± 4.6 | 6.4± 5.2 | 5.0± 3.5 | 6.3± 4.6 |
| Menopausal status |  |  |  |  |
| Pre-menopause | 24 (40.0) | 34 (69.4) | 88 (48.9) | 79 (65.8) |
| Post-menopause | 36 (60.0) | 15 (30.6) | 92 (51.1) | 40 (33.3) |
| unknown | 0 (0.0) | 0 (0.0) | 0 (0.0) | 1 (0.8) |
